# Tls1 regulates splicing of shelterin components to control telomeric heterochromatin assembly and telomere length

**DOI:** 10.1093/nar/gku842

**Published:** 2014-09-22

**Authors:** Jiyong Wang, Xavier Tadeo, Haitong Hou, Stuart Andrews, James J. Moresco, John R. Yates, Peter L. Nagy, Songtao Jia

**Affiliations:** 1 Department of Biological Sciences, Columbia University, New York, NY, USA; 2 Department of Pathology and Cell Biology, Columbia University College of Physicians and Surgeons, New York, NY, USA; 3 Department of Chemical Physiology, The Scripps Research Institute, La Jolla, CA, USA

## Abstract

Heterochromatin preferentially forms at repetitive DNA elements through RNAi-mediated targeting of histone-modifying enzymes. It was proposed that splicing factors interact with the RNAi machinery or regulate the splicing of repeat transcripts to directly participate in heterochromatin assembly. Here, by screening the fission yeast deletion library, we comprehensively identified factors required for telomeric heterochromatin assembly, including a novel gene *tls1^+^*. Purification of Tls1 and mass spectrometry analysis of its interacting proteins show that Tls1 associates with the spliceosome subunit Brr2. RNA sequencing analysis shows that the splicing of a subset of mRNAs are affected in *tls1Δ* cells, including mRNAs of shelterin components *rap1^+^* and *poz1^+^*. Importantly, replacing *rap1^+^* and *poz1^+^* with their cDNAs significantly alleviated heterochromatin defects of *tls1Δ* cells, suggesting that the missplicing of shelterin components is the cause of such defects, and that splicing factors regulate telomeric heterochromatin through the proper splicing of heterochromatin factors. In addition to its role in telomeric heterochromatin assembly, Tls1-mediated splicing of shelterin mRNAs also regulates telomere length. Given that its human homologue C9ORF78 also associates with the spliceosome and is overexpressed in multiple cancer cell lines, our results suggest that C9ORF78 overexpression might alter the proper splicing of genes during cancer progression.

## INTRODUCTION

Telomeres are protein-DNA complexes present at the ends of eukaryotic chromosomes (1–3). Telomeric DNA consists of double-stranded DNA (dsDNA) repeats followed by a 3′ single-stranded DNA (ssDNA) overhang, and is bound by a protein complex termed shelterin. Telomeres maintain genome integrity by protecting chromosome ends from being recognized as sites of DNA damage. They also prevent DNA replication-associated DNA loss through telomerase-mediated DNA sequence addition at chromosome ends. As most mammalian somatic cells do not express telomerase, telomeres gradually shorten upon each cell division. As a result, these cells can only divide a limited number of times and eventually enter senescence. In contrast, telomerase activity is high in stem cells and certain cancer cells, where it actively maintains telomere length and allows continued cell divisions (4). In single cell organisms, telomere length is generally maintained at a species-specific range (2). However, the mechanism that measures telomere length and controls telomerase activity is not well understood.

The fission yeast, *Schizosaccharomyces pombe*, is an excellent model organism for studying telomere structure and function due to highly conserved telomere organization and telomeric proteins, as well as the applicability of advanced genetic and biochemical tools. In this organism, the myb domain protein Taz1 (homologue of mammalian TRF1/2) binds the double-stranded telomeric DNA (5), while the Pot1/Tpz1 complex (homologue of mammalian POT1/TPP1 complex) binds the single-stranded telomeric overhang (6,7). Rap1 and Poz1 (similar to mammalian RAP1 and TIN2) form a bridge that connects these dsDNA and ssDNA binding proteins (6). Furthermore, Tpz1 associates with Ccq1, which recruits telomerase Trt1 to elongate telomeres (6,8–11). In fission yeast, telomeres are maintained at about 300 base pairs. Loss of *ccq1^+^* or *trt1^+^* results in gradual shortening of telomeres, and cells eventually enter senescence (8,9). On the other hand, loss of *taz1^+^*, *rap1^+^* or *poz1^+^* results in telomere elongation, suggesting that the normal function of these proteins is to negatively regulate telomere length (5,6,12–14). Pot1 and Tpz1 are also required for telomere end protection, and their loss results in rapid telomere loss, such that cells can only survive by circularizing all chromosomes (6,7).

Telomeres in diverse organisms form heterochromatin structures, which are condensed chromatin structures repressive to transcription and recombination (15). In fission yeast, heterochromatin formation requires the methylation of histone H3 lysine 9 (H3K9me) by histone methyltransferase Clr4 and the recruitment of heterochromatin protein 1 family protein Swi6. It also requires histone deacetylases Sir2 and SHREC (15). Heterochromatin is mainly present at pericentric regions and the silent mating-type region, in addition to telomeric regions. These regions share common pathways for the recruitment of histone modifying activities to establish heterochromatin. For example, all three regions contain a similar repetitive DNA element and the transcripts derived from these repeats are processed by the RNA interference (RNAi) machinery into small interfering RNAs (siRNAs), which can target the Clr4 complex to the nascent transcripts to initiate H3K9me at the repeat region (16). Heterochromatin formation can also be initiated by factors that specifically bind to each region. For example, the stress-activated CREB family proteins Atf1/Pcr1 recruit heterochromatin factors to the silent mating-type region (17,18), and shelterin is responsible for the recruitment of heterochromatin assembly machinery to telomere repeats (19,20). Therefore, identification of factors that specifically affect telomeric heterochromatin assembly without affecting heterochromatin assembly at other locations might allow identification of novel factors that regulate shelterin function. For example, shelterin component Taz1 was originally identified through a yeast one-hybrid screen for factors that bind to telomeric DNA and was found to regulate both telomeric silencing and telomere length (5). It was also identified independently through a genetic screen for factors that affect telomeric silencing (12).

In this study, through a screen for mutants that affect silencing of a telomeric reporter, we found that an uncharacterized gene, *tls1^+^*, is required for telomeric heterochromatin assembly as well as telomere length control. We further found that Tls1 associates with spliceosome component Brr2 and regulates the proper splicing of mRNAs, including shelterin components *rap1^+^* and *poz1^+^* to indirectly regulate telomeric heterochromatin and telomere length.

Recently, splicing factor mutants were identified in screens that affect heterochromatin assembly at pericentric regions (21–23). As the transcripts derived from repeat regions contain introns, an attractive hypothesis is that splicing factors are directly involved in heterochromatin assembly through binding or splicing of non-coding RNAs (21–23). However, our recent analyses suggest that splicing factors affect pericentric heterochromatin assembly by regulating the proper splicing of mRNAs of RNAi factors rather than through a direct role in the heterochromatin assembly process (24). Our results here further support the idea that splicing factors regulate heterochromatin assembly indirectly by regulating the proper splicing of heterochromatin assembly factors.

Altogether, our results provide a comprehensive analysis of telomeric heterochromatin assembly factors and identify a novel gene that regulates both telomeric heterochromatin assembly and telomere length. The human homologue of Tls1, C9ORF78, is overexpressed in multiple cancer cell lines (25). Our results suggest that overexpression of C9ORF78 might affect proper mRNA splicing to affect cancer progression.

## MATERIALS AND METHODS

### Genetic screen of the fission yeast deletion library

Query strain (Ch16-*natMX6*) was constructed by inserting the *natMX6* cassette at the *ade6* locus on Ch16 (26). It was mated with the fission yeast deletion library arrayed in 384 strains/plate format with the aid of the Singer RoToR HDA pinning robot as previously described (27). After mating and selection, the resulting haploid cells containing Ch16 and an individual gene deletion were pinned to medium without uracil to measure cell growth.

### Fission yeast strains and genetic analyses

Detailed genotypes of strains used are listed in Supplementary Table S1. Strains containing *tls1Δ*, Brr2-myc, Brr2-Flag, Rap1-Flag, Poz1-myc, Poz1-GFP, Pot1-myc and Pot1-GFP were constructed by a polymerase chain reaction (PCR)-based module method. *tls1-1* was derived from the Bioneer fission yeast deletion library, verified via PCR, and backcrossed. The *pREP41-Flag-Tls1^+^* plasmid was constructed by inserting a PCR product containing *Tls1^+^* coding region into BamHI and XmaI sites. *c-rap1* was constructed with *rap1* cDNA combined with a Flag tag and a *natMX6* cassette at the endogenous *rap1^+^* locus and *c-poz1* was constructed with *poz1* cDNA combined with a myc tag and a *kanMX6* cassette at the endogenous *poz1^+^* locus. Genetic crosses were used to construct all other strains. For serial dilution plating assays, 10-fold dilutions of mid-log-phase culture were plated on the indicated medium and grown for 3 days at 30°C.

### RNA analyses

Total cellular RNA was isolated from log-phase cells using MasterPure yeast RNA purification kit (Epicentre) according to the manufacturer's protocol. Quantification with quantitative reverse transcriptase-PCR (qRT-PCR) was performed with Power SYBR Green RNA-to-CT one-step Kit (Applied Biosystems). RNA serial dilutions were used as templates to generate the standard curve of amplification for each pair of primers, and the relative concentration of target sequence was calculated accordingly. An *act1* fragment served as reference to normalize the concentration of samples. The concentration of each target gene in wild type was arbitrarily set to 1 and served as references for other samples. Oligos used are listed in Supplementary Table S2.

For RNA-seq, purified RNA was prepared by TruSeq Stranded Total RNA Kit (Illumina), which includes rRNA depletion and chemical fragmentation. Index adapters were added to allow for multiplexing. Paired-end sequencing with 100 bp read lengths was performed on Illumina HiSeq. Mapping was performed with the Tuxedo Suite consisting of Bowtie, TopHat and Cufflinks. A total of 31 789 605 mapped reads were obtained for *tls1Δ* and 20 208 005 mapped reads were obtained for wild type. For the dot plot, exon–exon junction ratios were filtered to remove: (i) junctions which mapped zero times in any sample (possible mapping noise), (ii) junctions whose read counts in wild type and *tls1Δ* were less than 0.5 per million reads (to avoid randomness due to small sample sizes).

### Chromatin immunoprecipitation (ChIP) analysis

ChIP analyses with H3K9me2 antibody (Abcam 1220) were performed as described previously (28). Quantification with qPCR was performed with Maxima SYBR Green/ROX qPCR Master Mix (Thermo). DNA serial dilutions were used as templates to generate a standard curve of amplification for each pair of primers, and the relative concentrations of target sequence and a control *act1* sequence in ChIP and whole cell extract (WCE) samples were calculated accordingly. The final enrichment was calculated as [(ChIP target)/(WCE target)]/[(ChIP *act1*)/(WCE *act1*)]. Oligos used are listed in Supplementary Table S2.

### Protein purification

Exponentially growing yeast cells were harvested, washed with 2×HC buffer (300 mM HEPES-KOH pH 7.6, 2 mM EDTA, 100 mM KCl, 20% glycerol, 0.1% NP-40, 2 mM DTT and protease inhibitor cocktail (Roche)) and frozen in liquid nitrogen. Crude cell extracts were prepared by vigorously blending frozen yeast cells with dry ice in a household blender, followed by sonication and incubation with 30 ml 1×HC buffer containing 250 mM KCl for 30 min. The lysate was cleared by centrifugation at 82 700 × *g* for 3 h. The supernatants were incubated with 150 μl of Flag antibody agarose beads, washed eight times with 1×HC containing 250 mM KCl, then two times with the same buffer without NP-40. For mass spectrometry analysis, bound proteins were eluted with 2 × 400 μl of 50 mM Tris pH 7.5, 5% SDS, 5% glycerol and 50 mM DTT for Cwf14-Myc purifications and with 200 mg/ml 3×Flag peptides for Flag-tagged protein purifications. For co-immunoprecipitation analyses, bound proteins were separated by sodium dodecyl sulphate-polyacrylamide gel electrophoresis (SDS-PAGE), followed by western blot analyses with Myc (Santa Cruz Biotechnology, sc-789) and Flag (Sigma, F7425) antibodies.

### MudPIT analysis

Protein pellets were dissolved in buffer (8 M urea, 100 mM Tris pH 8.5), reduced with TCEP (Tris[2-Carboxyethyl]-Phosphine Hydrochloride) and alklyated with chloroacetamide. After dilution of urea to 2 M, proteins were digested with trypsin. Digested peptides were analyzed by LC/LC/MS/MS using an Linear Trap Quadropole (LTQ) mass spectrometer. Multidimensional chromatography was performed online with 6 salt steps (29). Tandem mass spectra were collected in a data-dependent manner with up to 5 ms2 scans performed for each initial scan (m/z range 300–2000). The search program, Prolucid (30), was used to match data to a fission yeast protein database. Peptide identifications were filtered using the DTASelect program (31).

### Southern blot analyses

Genomic DNAs were extracted, digested by EcoRI and separated on 1% agarose gel. Then DNAs in the agarose gel were transferred onto positively charged Nylon membrane followed by hybridization with ^32^P-labeled telomere probes and autoradiography as described previously (32).

### Yeast two-hybrid assay

Full-length *tls1^+^* was cloned into XmaI/BamHI site of pGBT9 (Clontech) to generate fusion with the GAL4 DNA-binding domain. Full-length *brr2^+^* was cloned into PstI/BglII site of pGAD424 (Clontech) to generate fusion with the GAL4 activation domain. Both plasmids were transformed into the budding yeast strain pJ69-4A, and transformants were selected on medium lacking tryptophan and leucine to maintain both plasmids. The interaction of the two proteins was indicated by the activation of a *HIS3* reporter, allowing growth on medium lacking histidine.

## RESULTS

### Identification of Tls1 as a factor required for telomeric heterochromatin assembly

**Figure 1.**
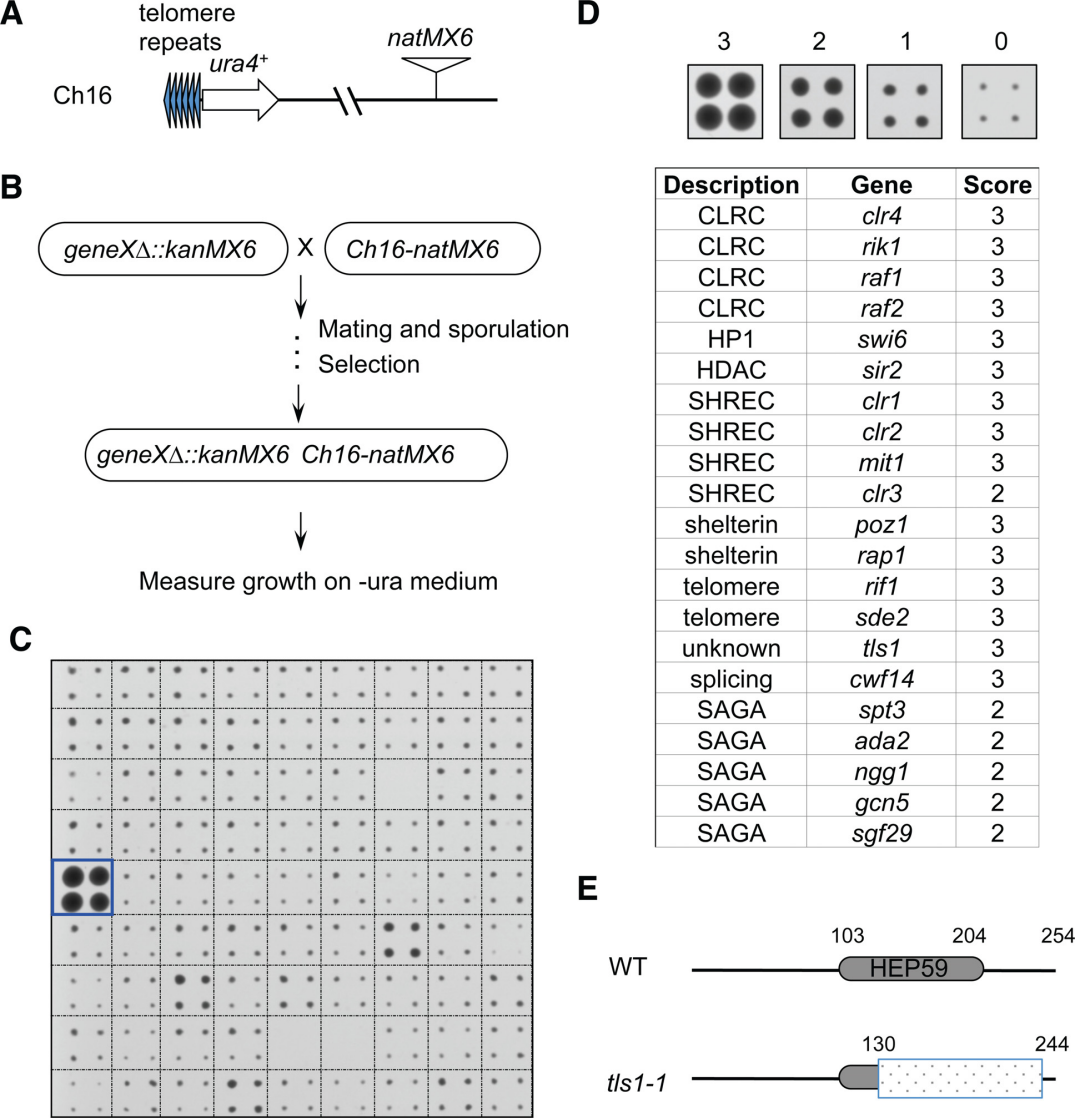
A genetic screen for non-essential genes required for telomeric heterochromatin silencing in *S. pombe*. (A) Schematic diagram of the *Tel::ura4^+^-natMX6* reporter. (B) Workflow to introduce *TEL::ura4^+^* into the deletion library. (C) A representative image of cells grown on medium without uracil (-ura). Box indicates the position of *tls1-1*. (D) List of identified mutants affecting telomeric silencing. Hit colonies were assigned scores between 1 and 3, as indicated, 0 indicates background growth. (E) Schematic diagram of Tls1, the position of HEP59 domain was indicated. Shaded box indicates region deleted in *tls1-1*.

There are three pathways that function at native telomeres to regulate heterochromatin assembly: telomeric repeats recruit shelterin, which in turn recruits Clr4; a repetitive DNA element within the *tlh1^+^* gene mediates the recruitment of Clr4 through the RNAi pathway; telomere-associated sequences (TAS) regulate heterochromatin assembly through an unknown mechanism (19). Due to such redundancy, the effects of shelterin or RNAi mutations on heterochromatin assembly at native telomeres are either very mild or undetectable. In contrast, silencing of a *ura4^+^* reporter gene inserted near telomeric repeats of a mini-chromosome Ch16 (*TEL::ura4^+^*) (33) (Figure 1A), which lacks *tlh1^+^* and TAS, is critically dependent on shelterin. Thus, *TEL::ura4^+^* is a very sensitive reporter for the identification of mutants that affect shelterin function.

We modified Ch16 with a *natMX6* cassette (Figure 1A) (34), which allows selection by nourseothricin. We then introduced the modified mini-chromosome into the fission yeast deletion library using robot-aided genetic crosses (Figure 1B) (27). After selecting haploid progenies containing both the mini-chromosome and individual gene deletions, cells were assayed for silencing of *TEL::ura4^+^* by measuring growth on medium without uracil (Figure 1C). We identified a number of mutants known to affect telomere silencing, including shelterin components (*poz1Δ* and *rap1Δ*) (14,35), telomere silencing factors (*rif1Δ* and *sde2Δ*) (14,36), as well as mutations in general heterochromatin assembly factors, such as CLRC (*clr4Δ*, *rik1Δ*, *raf1Δ* and *raf2Δ*), SHREC (*clr1Δ*, *clr2Δ*, *mit1Δ* and *clr3Δ*), *swi6Δ* and *sir2Δ* (37), demonstrating the effectiveness of our screen (Figure 1D). Other known factors, such as *taz1Δ* and *chp2Δ*, were either incorrect or absent in our library, and therefore were not identified as hits.

Most interestingly, we identified a mutation in a novel gene, *SPAC1D4.01*, which showed strong defects in *TEL::ura4^+^* silencing. Accordingly, we named the gene *tls1* (telomere length and silencing 1). Its mammalian homologue is the uncharacterized gene *C9ORF78*, which is overexpressed in a number of cancer cell lines (25). The main feature of its protein product is a hepatocellular carcinoma-associated antigen 59 (HEP59) domain, which is conserved across species (Supplementary Figure S1). Because the *tls1* mutation in the Bioneer deletion library only removes the majority of the HEP59 domain (deletion of amino acids 130–244), and still retains a large portion of its open reading frame, we named it *tls1-1* (Figure 1E). Our screen also identified mutations in a number of components of SAGA (*gcn5Δ*, *ada2Δ*, *ngg1Δ*, *sgf29Δ* and *spt3Δ*) (Figure 1D), which is a histone acetyltransferase complex involved in diverse aspects of chromatin metabolism (38). However, the effect of SAGA mutants were weaker compared to that of *tls1-1*, so we focused our analysis on Tls1 function, and the characterization of SAGA in telomere silencing will be described elsewhere. We also identified *meu6Δ* as a strong hit in our screen (category 3). However, our independently constructed *meu6Δ* (which removed the entire open reading frame (ORF) of *meu6^+^*) has no effect on *TEL::ura4^+^* silencing (our unpublished data). Given that *meu6^+^* is next to *clr4^+^*, the *meu6Δ* in the Bioneer library is most likely an imprecise deletion that affected the function of *clr4^+^*.

### Tls1 is required for telomeric heterochromatin assembly

**Figure 2.**
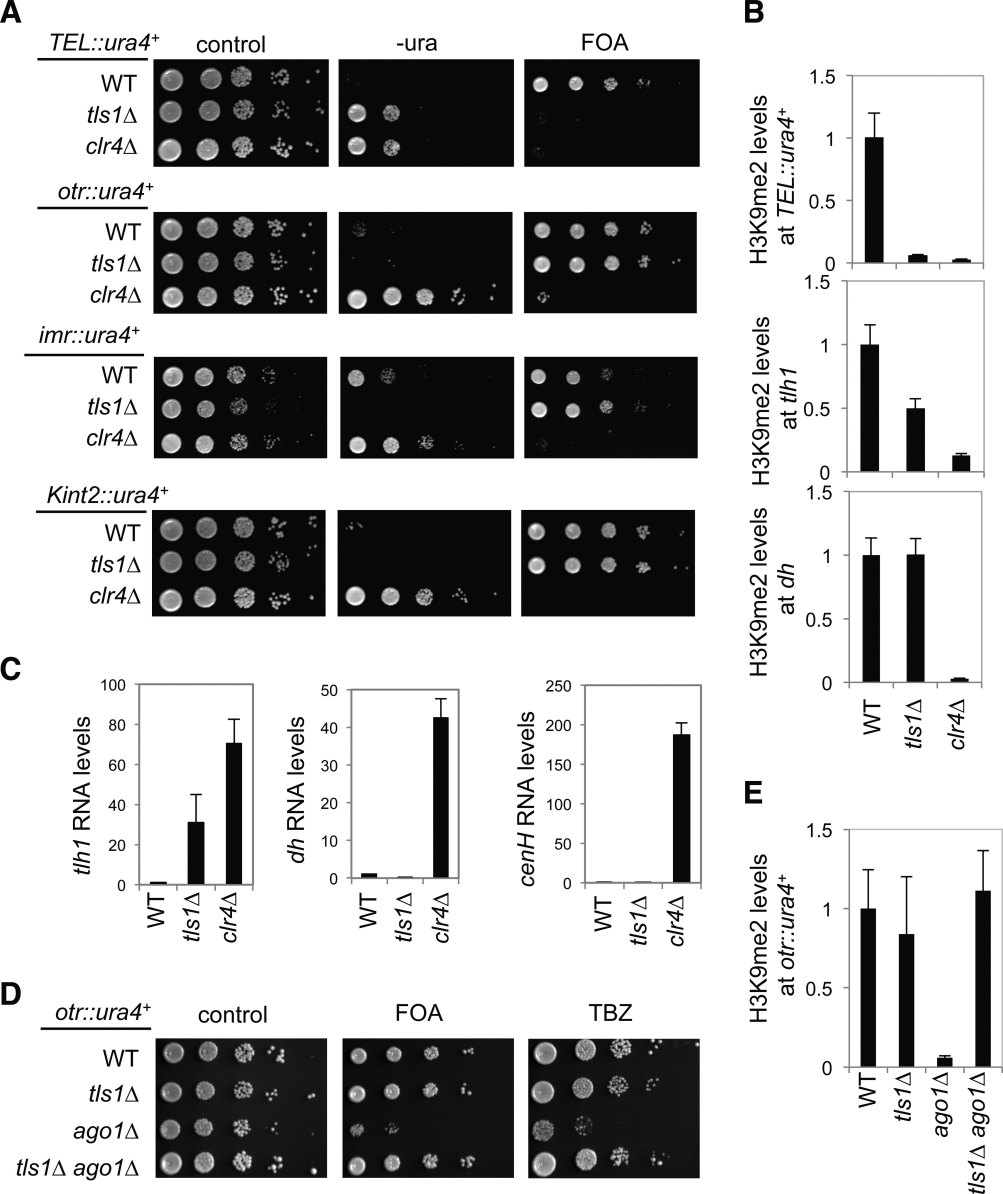
Tls1 is specifically required for telomeric heterochromatin assembly. (A and D) Serial dilution analyses to measure the expression of *ura4^+^* reporter genes and sensitivity to TBZ. (B and E) ChIP analyses of H3K9me2 levels at *ura4^+^* reporter genes, normalized to *act1* gene. Error bars represent standard deviation of three experiments. (C) qRT-PCR analyses of transcripts derived from repeat elements, *tlh1* for subtelomeric region, *dh* for pericentric region and *cenH* for mating type locus, normalized to *act1*. Wild-type levels were set to 1. Error bars represent standard deviation of three experiments.

To determine whether the telomeric silencing defect results from loss of Tls1 or a dominant-negative function of *tls1-1*, we generated a complete deletion of *tls1^+^* (*tls1Δ*). We found that *tls1Δ* also severely affected the silencing of *TEL::ura4^+^* as indicated by serial dilution analysis of cells on medium without uracil and on medium containing 5-fluoroorotic acid (FOA), which is toxic to cells expressing Ura4 (Figure 2A). Moreover, heterochromatin hallmark H3K9me2 was reduced at *TEL::ura4^+^* to a level similar to that of *clr4Δ* (Figure 2B). In addition, H3K9me2 levels were also significantly reduced, although not abolished, at the *tlh1^+^* locus, which is embedded within subtelomeric heterochromatin (Figure 2B). Consistently, *tls1Δ* showed increased accumulation of transcripts of *tlh1^+^* (Figure 2C). The milder effect of *tls1Δ* at endogenous subtelomeric regions is likely due to the existence of redundant heterochromatin assembly pathways. Interestingly, *tls1Δ* slightly strengthened reporter silencing at the pericentric region (*otr::ura4^+^* and *imr::ura4^+^*) or at the silent mating-type locus (*Kint2::ura4^+^*) as indicated by reduced growth on medium without uracil (Figure 2B). Although we did not observe significant changes in H3K9me2 levels at pericentric *dh* repeats in *tls1Δ* cells (data not shown), transcription of pericentric *dh* repeats or mating type region *cenH* are slightly lower than wild type (Figure 2C and Supplementary Figure S2), indicating that *tls1Δ* enhances silencing at non-telomeric locations. As with *tls1Δ*, we found that *tls1-1* resulted in loss of silencing at *TEL::ura4^+^*, but not at *otr::ura4^+^* or *Kint2::ura4^+^* (Supplementary Figure S3), suggesting that *tls1-1* is a loss-of-function mutant.

The formation of pericentric heterochromatin requires the RNAi pathway (39). Mutations of RNAi factors, such as *ago1Δ*, result in the loss of silencing of a pericentric heterochromatin reporter gene (*otr::ura4^+^*). We previously performed high-throughput screens for mutants that bypass the requirement of the RNAi machinery for pericentric heterochromatin assembly and identified a number of factors that affect telomere silencing (20). These mutants result in the release of Swi6 protein from telomeres to strengthen RNAi-independent heterochromatin assembly pathways at pericentric regions (20). Interestingly, *tls1-1* was also consistently identified in all such screens. We confirmed that *tls1Δ ago1Δ*, *tls1-1 ago1Δ*, *tls1-1 dcr1Δ* cells all restored silencing of *otr::ura4^+^* (Figure 2D and Supplementary Figure S3). Moreover, ChIP analysis showed wild-type levels of H3K9me2 at centromeric reporter in *tls1Δ ago1Δ* cells (Figure 2E). RNAi mutant cells are also sensitive to microtubule poison thiabendazole (TBZ) due to failure to recruit cohesin to pericentric regions (40,41). The *tls1Δ ago1Δ* cells are no longer sensitive to TBZ, indicating that proper function of pericentric heterochromatin is also restored (Figure 2D).

Altogether, these results suggest that Tls1 is specifically required for heterochromatin assembly at telomeric regions, but not for heterochromatin assembly at centromeres or the silent mating-type region. Therefore, Tls1 is more likely to be a telomere regulator than a component of the core heterochromatin assembly machinery.

### Tls1 is required for telomere length control

**Figure 3.**
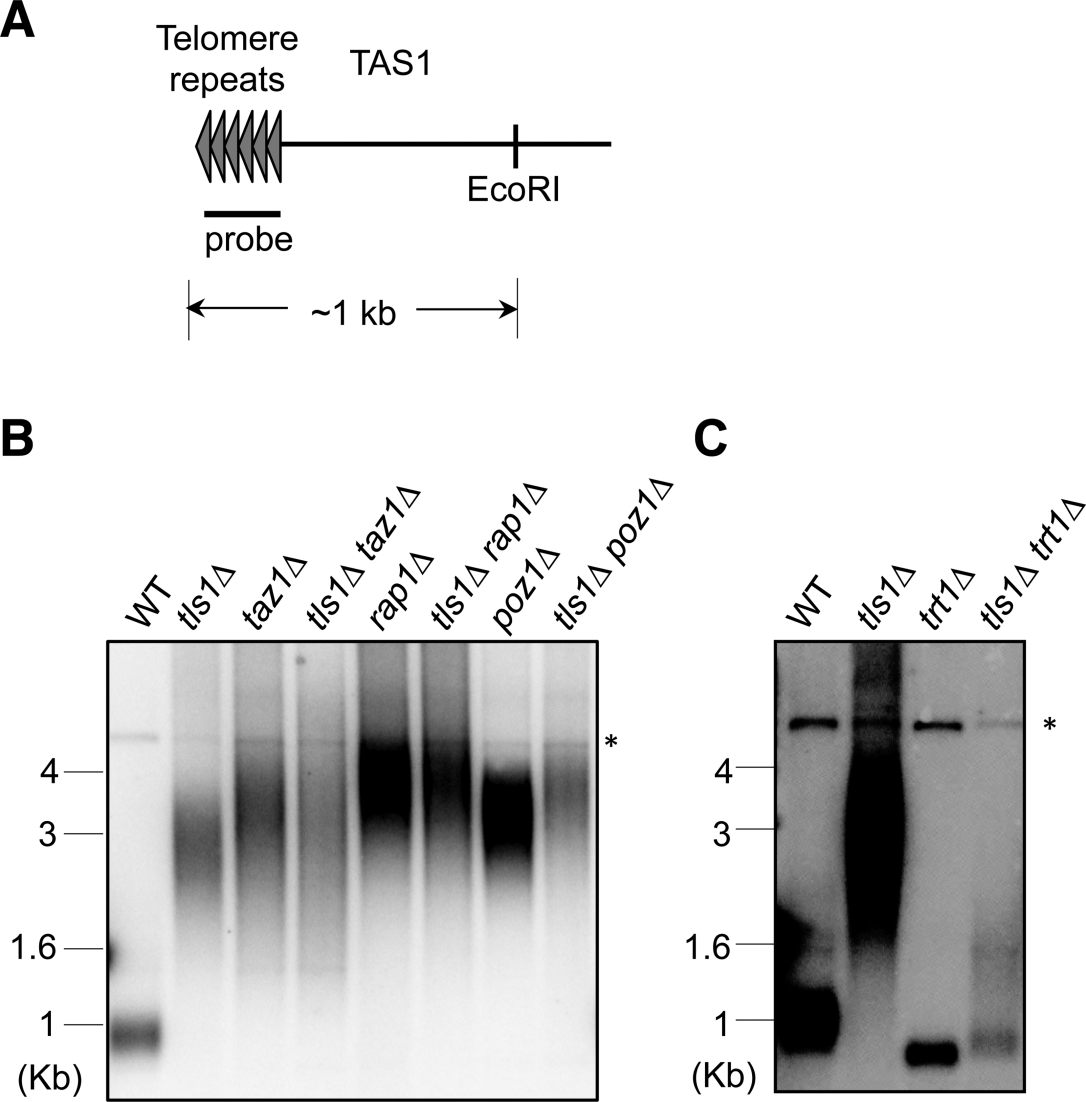
Tls1 regulates telomere length. (A) Schematic diagrams of native telomeres. The positions of probe and EcoRI enzymatic site are indicated. (B and C) Genomic DNAs were isolated, digested by EcoRI, and Southern blot analyses were performed with a telomere probe. Asterisk represents a non-specific band serving as a loading control.

In fission yeast, telomere length is maintained at ∼300 base pairs (Figure 3A). Mutations in a number of shelterin components that affect telomere silencing, such as in *taz1Δ*, *rap1Δ* and *poz1Δ*, also result in telomere elongation (5,6,13,14). If Tls1 regulates the function of these shelterin components, we expect loss of Tls1 to affect telomere length as well. Indeed, a screen for mutants that affect telomere length showed that *tls1-1* cells significantly elongated telomeres (42). Our Southern blot analyses with a probe specific for telomeric repeats also showed that *tls1Δ* cells have much longer telomeres, to an extent similar to those of *taz1Δ*, *rap1Δ* and *poz1Δ* (Figure 3B). Moreover, *tls1Δ taz1Δ*, *tls1Δ rap1Δ* and *tls1Δ poz1Δ* cells all showed similar levels of telomere elongation (Figure 3B). Telomere elongation in *taz1Δ* and *poz1Δ* cells depends on telomerase Trt1, and telomere length is reduced to below wild*-*type levels in *taz1Δ trt1Δ* and *poz1Δ trt1Δ* cells (6,43). Similarly, in *tls1Δ trt1Δ* cells, telomere length is reduced to below wild-type levels (Figure 3C), suggesting that the long telomeres in *tls1Δ* cells are mediated by telomerase. The *tls1Δ trt1Δ* cells used were freshly generated, thus are unlikely to circularize their chromosomes. Confirming such an idea, PCR analysis showed these cells are not sensitive to MMS and has robust TAS1 signal, which are different from cells circular chromosomes (data not shown). Altogether, these results suggest that Tls1 functions in the same pathway as Taz1, Rap1 and Poz1.

### Tls1 associates with the spliceosome

**Figure 4.**
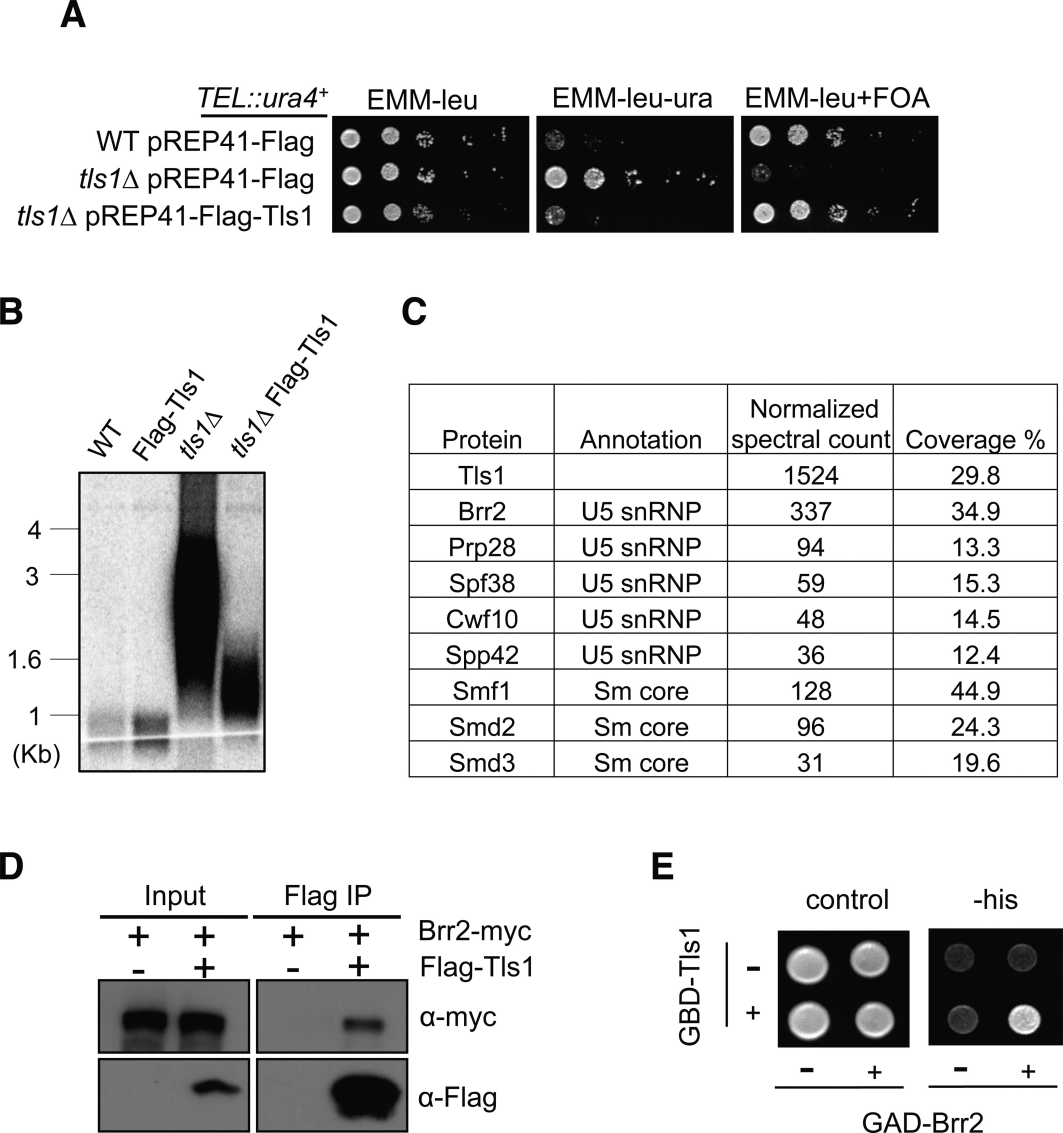
Tls1 associates with the spliceosome. (A) Flag-Tls1 is functional in telomere silencing as indicated by serial dilution analysis to measure the expression of *TEL::ura4^+^*. (B) Flag-Tls1 is functional in telomere length control as indicated by Southern blot analysis with a telomere probe. (C) Mass spectrometry analyses of purified Flag-Tls1 complex. (D) Co-immunoprecipitation analysis of Flag-Tls1 and Brr2-myc. The whole cell extracts were incubated with Flag-agarose beads to pull down Flag-tagged Tls1. Flag and myc antibodies were used to perform western blot analyses. (E) Tls1 interacts with Brr2 in a yeast two-hybrid assay. Tls1 was fused with Gal4 DNA-binding domain and Brr2 was fused with an activation domain. Interaction between Tls1 and Brr2 resulted in the activation of a *HIS3* reporter gene, allowing cells to grow in the absence of histidine.

To further analyze the function of Tls1, we attempted to generate epitope-tagged versions of Tls1 at the endogenous *tls1^+^* locus. However, we were unable to obtain tagged versions of this protein with detectable western signal (data not shown). We thus generated a plasmid that expresses N-terminal Flag-tagged Tls1 under the control of an inducible *nmt41* promoter. Under induced conditions, the plasmid-born *tls1^+^* rescued the silencing defects of *TEL::ura4^+^* and also significantly reduced telomere elongation associated with *tls1Δ* (Figure 4A and B). The incomplete rescue of telomere length defects by plasmid born Flag-Tls1 might be attributed to its higher expression levels or that Flag-Tls1 is not fully functional.

We then performed affinity purification of Flag-Tls1, and subjected the purified complex to mass spectrometry analyses (Figure 4C and Supplementary Table S3). We found that Tls1 associated with Brr2, which is part of the U5-snRNP involved in multiple steps of mRNA splicing. A number of other U5-snRNP components and Sm core proteins were also identified in our Tls1 purification, although at much lower quantities as indicated by normalized spectral counts. We constructed a C-terminal myc-tagged version of Brr2 at its endogenous chromosome location. Co-immunoprecipitation analyses showed that Flag-Tls1 indeed associates with Brr2-myc (Figure 4D). We note that all the experiments are performed with plasmid born Flag-Tls1, which are expressed at higher levels than those of endogenous Tls1 and might not reflect the situation in wild-type cells. Furthermore, Brr2 interacts with Tls1 in a yeast two-hybrid assay (Figure 4E), suggesting that these two proteins might directly interact with each other.

### Tls1 regulates spliceosome assembly and proper splicing of mRNAs

The function of the spliceosome is best characterized in human and budding yeast. The spliceosome is composed of U1, U2, U4, U5 and U6 small nuclear RNAs (snRNAs) that form small nuclear ribonucleic particles (snRNPs), as well as many additional factors, such as Sm proteins required for snRNP assembly (44). The spliceosome is assembled on the introns of mRNAs in a series of steps. Initially, the U1 and U2 snRNPs bind to the 5′ splice site and branch point on a pre-mRNA, respectively, to form the pre-spliceosome. The U4/U6.U5 snRNPs are then recruited to form the pre-catalytic spliceosome. The addition of the Prp19 complex (also known as NineTeen Complex) and the release of U1 and U4 snRNPs result in structural rearrangement leading to the formation of an activated spliceosome, which catalyzes the cleavage of 5′ splice site and fusion with the branch point to form a lariat structure. The 3′ intron cleavage is coupled with exon ligation, resulting in the release of mature mRNA, the disassembly of spliceosome and recycling of the components for the next round of splicing reaction. Brr2 is a DEAD/DEAH-box motif ATPase that is a component of the U5 snRNP. It is required for the unwinding of U4/U6 to allow the refolding of U6 with U2 for spliceosome activation (45–48). Brr2 is also required for conformational rearrangement during the splicing reaction (49) as well as the unwinding of U2/U6 at the end of splicing to allow the disassembly of spliceosome (50).

In fission yeast, the most stable and abundant form of endogenous spliceosome complexes are late stage U5.U2/U6 complexes (51–54). Proteomic analysis show that Brr2 weakly associates this late stage spliceosome (54). However, biochemical analyses of Brr2 showed that it forms a range of complexes, consistent with the idea that Brr2 functions at multiple steps during spliceosome assembly (55).

To examine whether Tls1 regulates the composition of the spliceosome, we performed affinity purification of Brr2-Flag or Cwf14-myc in the presence or absence of Tls1 and then identified interacting proteins by mass spectrometry (Supplementary Tables S4 and S5). However, we only detected minor changes in spliceosome composition in the absence of Tls1, suggesting that Tls1 does not have a major impact on spliceosome assembly.

**Figure 5.**
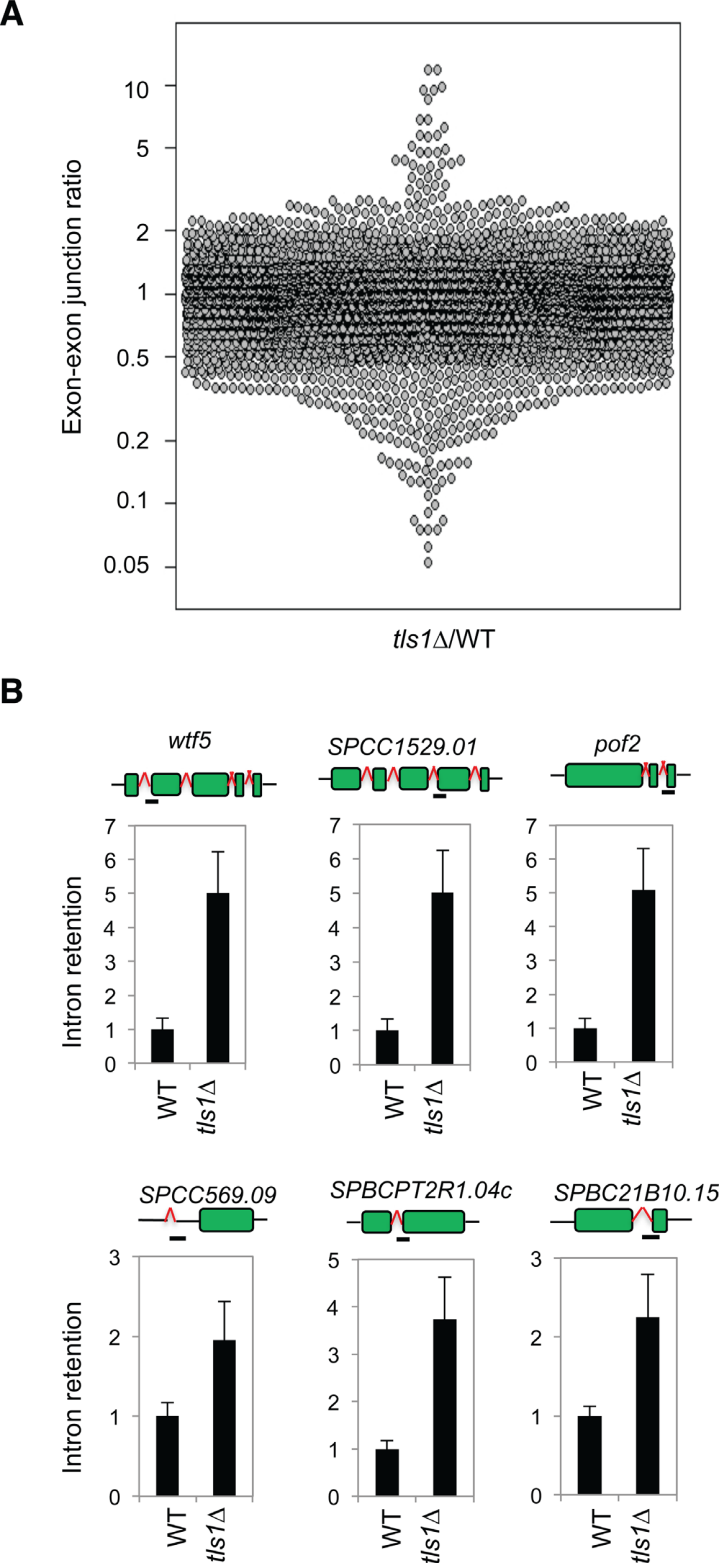
Tls1 is required for proper mRNA splicing. (A) Ratios of exon–exon junction counts from RNA-seq for each junction in the indicated samples. A ratio of 1 indicates no difference. The bottom tail indicates missplicing. (B) qRT-PCR analyses of intron retention levels, normalized to *act1*. Wild-type levels were set to 1. Bars represent PCR fragment amplified.

To examine the effect of Tls1 on mRNA splicing, we performed sequencing of total RNAs isolated from wild-type and *tls1Δ* cells. In fission yeast, 43% of genes contain introns, indicating the prevalence of splicing in this organism. The majority of introns were properly processed, even in *tls1Δ* cells, suggesting that Tls1 only selectively affected the activity of the spliceosome (Supplementary Table S6 and Figure 5A). Nonetheless, *tls1Δ* cells showed more than 2-fold increase in intron retention of 7.3% introns, suggesting that it selectively regulates mRNA splicing. We note that since only a single sequencing reaction was performed for each sample, sequences with low counts are more likely to show random variations. Therefore, we performed qRT-PCR analyses of a number of highly misspliced introns and the results confirmed that they are indeed misspliced (Figure 5B), suggesting that Tls1 indeed regulates mRNA splicing. A number of introns also showed less intron retention. However, closer examination showed that many of these are highly abundant non-coding RNAs, such as rRNAs, which might have mapping issues. qRT-PCR analyses of a few genes of this group showed similar intron retention level as wild type (data not shown).

### Tls1 regulates the proper splicing of shelterin components

**Figure 6.**
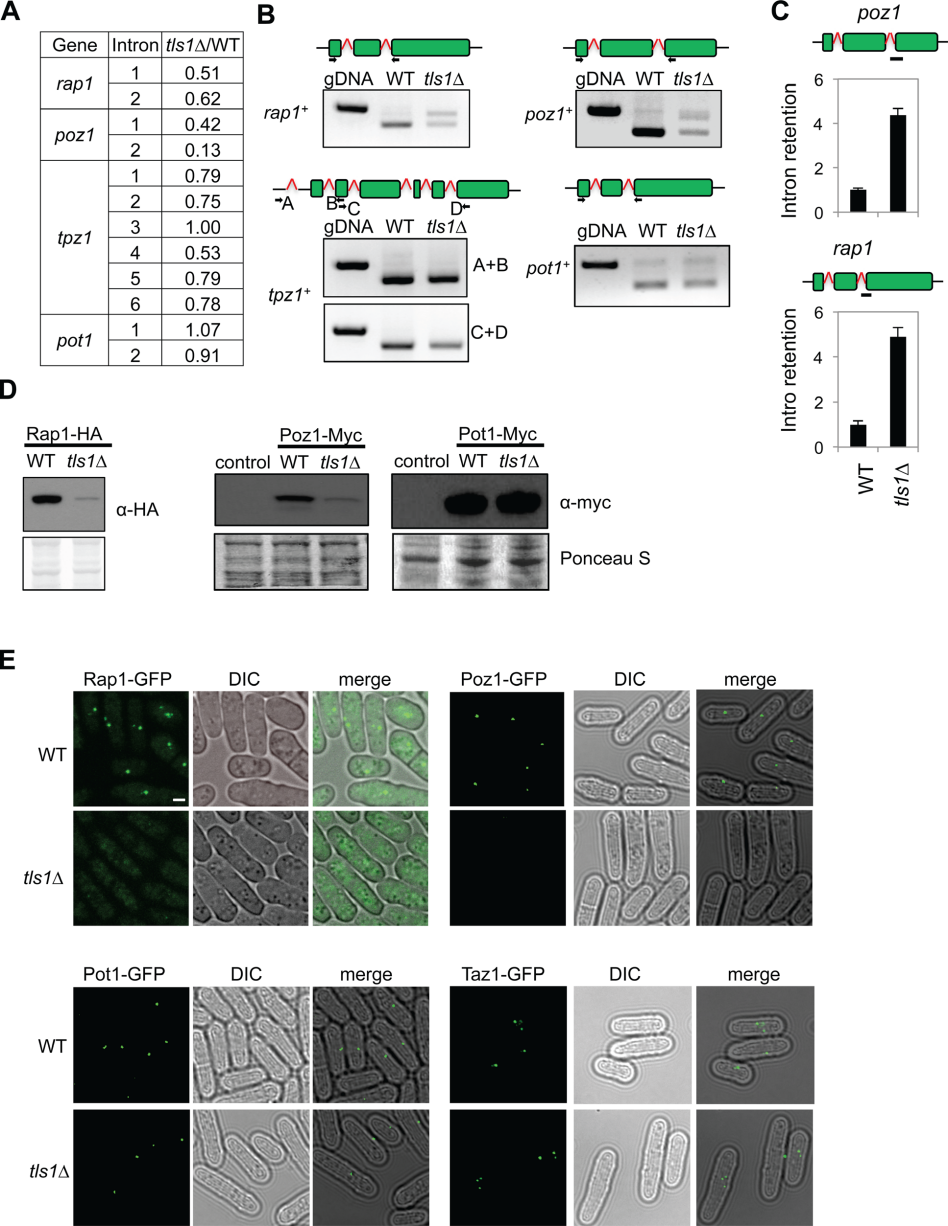
Tls1 regulates the proper splicing of shelterin components. (A) List of number of introns present in the genes of telomere proteins. (B) RT-PCR analyses of RNA with primers flanking introns. Genomic DNA (gDNA) was used in a control PCR reaction to indicate the position of unspliced form. The structures of the genes are indicated on top and boxes represent exons. The primer positions were labeled. (C) qRT-PCR analyses of intron retention levels, normalized to *act1*. Wild-type levels were set to 1. Bars represent PCR fragment amplified. (D) Western blot analyses of shelterin protein levels. Ponceau S staining was used as the loading control. (E) Live cell imaging of cells expressing GFP-tagged versions of shelterin. Scale bar, 1 μm.

The similarity of phenotypes between *tls1Δ* and shelterin mutants, such as *poz1Δ*, *rap1Δ* and *taz1Δ*, prompted us to test whether *tls1Δ* resulted in splicing defects of shelterin components. Among shelterin components and factors that affect telomere silencing, only *rap1^+^*, *poz1^+^*, *tpz1^+^* and *pot1^+^* have introns, and our RNA-seq analysis showed that the splicing of *rap1^+^*, *poz1^+^* and *tpz1^+^* were likely affected (Figure 6A). RT-PCR analyses of total RNAs with primers flanking introns showed that both *rap1^+^* and *poz1^+^* RNAs were misspliced in *tls1Δ* cells, whereas the splicing of *tpz1^+^* and *pot1^+^* appears normal (Figure 6B and C). Furthermore, western blot analyses showed that both Rap1-HA and Poz1-myc protein levels were significantly reduced in *tls1Δ* cells (Figure 6D). We did not detect truncated forms of Rap1-HA or Poz1-myc in *tls1Δ* cells in our western blot analyses. However, since these proteins are C-terminally tagged, we could not rule out the possibility that truncated forms of these proteins are present in *tls1Δ* cells that function in a dominant-negative fashion to affect telomere silencing and telomere length control.

The reduction of Rap1 and Poz1 protein levels in *tls1Δ* cells was further corroborated by reduced Rap1-GFP and Poz1-GFP signals in live cells (Figure 6D). In contrast, the localization of Taz1-GFP or Pot1-GFP to telomeres was not significantly affected in *tls1Δ* cells. Thus, loss of Tls1 specifically affected the splicing of *rap1^+^* and *poz1^+^*, which is consistent with the phenotypes of *rap1Δ* and *poz1Δ* in telomere silencing and telomere length control.

**Figure 7.**
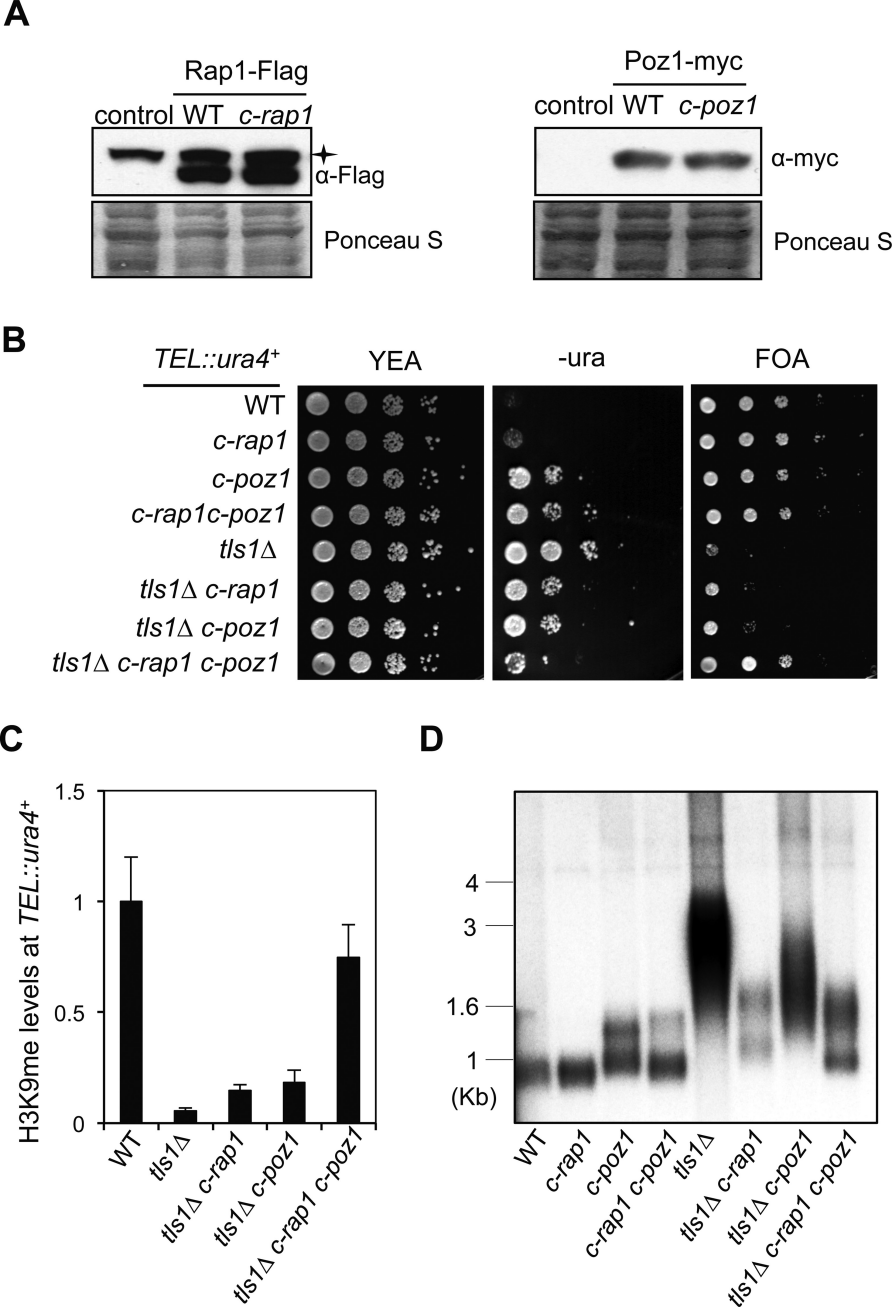
Introducing cDNAs of shelterin is sufficient to restore telomeric heterochromatin and proper telomere length in *tls1Δ* cells. (A) Western blot analysis of shelterin protein levels. Asterisk indicates a non-specific band. (B) Serial dilution analyses to measure *TEL::ura4^+^* reporter gene expression. (C) ChIP analyses of H3K9me2 levels at *TEL::ura4^+^*, normalized to *act1*. Error bars represent standard deviation of three experiments. (D) Southern blot analyses of telomere length with a telomere probe.

To further confirm that missplicing of *rap1^+^* and *poz1^+^* is the main effect of *tls1Δ* on telomere silencing and telomere length, we replaced the genomic DNA of *rap1^+^* and *poz1^+^* with their corresponding cDNAs at their endogenous chromosome locations (*c-rap1* and *c-poz1*, respectively). Both Rap1 and Poz1 proteins are expressed at normal levels from these cDNA constructs as indicated by western blot analyses (Figure 7A). Cells containing *c-rap1* did not show any silencing defects of *TEL::ura4^+^*, and the telomere length was similar to that of wild type, although cells with *c-poz1* showed mild defects in telomere silencing and slightly elongated telomeres. Introducing either *c-rap1* or *c-poz1* into *tls1Δ* cells resulted in slight alleviation of telomeric silencing and telomere length defects, consistent with *tls1Δ* resulting in partial loss of function of both Rap1 and Poz1. Most importantly, when both *c-rap1* and *c-poz1* were introduced into *tls1Δ* cells, telomere silencing and telomere length defects were more significantly rescued (Figure 7B–D). These results confirmed that Tls1regulates the proper splicing of Rap1 and Poz1 to control telomeric heterochromatin assembly and telomere length.

## DISCUSSION

In this study, we identified an uncharacterized gene *tls1^+^* required for telomere silencing and telomere length control. We further showed that Tls1 associates with spliceosome component Brr2 and regulates the proper splicing of mRNAs, including those of *rap1^+^* and *poz1^+^*. Moreover, introducing cDNA versions of *rap1^+^* and *poz1^+^* bypassed the requirement of Tls1 for telomere silencing and telomere length control.

In fission yeast, the RNAi machinery processes transcripts derived from repetitive DNA elements into siRNAs, which target CLRC to initiate histone H3K9 methylation (16). In addition, splicing factors are required for RNAi-mediated heterochromatin assembly (21–23). It was proposed that splicing factors regulate heterochromatin assembly by forming a platform for the assembly of heterochromatin factors on the nascent transcript or by regulating splicing of the repeat transcripts. We have recently performed a deletion library-based screen for factors required for pericentric heterochromatin assembly and identified a number of splicing factor mutants (56). Sequencing of total RNAs from one of the strongest mutants, *cwf14Δ*, shows that the splicing of a subset of genes, including many RNAi factors, is affected. Importantly, introducing cDNA versions of several RNAi factors significantly rescues silencing defects of *cwf14Δ* at pericentric regions, suggesting that the major function of splicing factors in heterochromatin assembly is to regulate the proper splicing of RNAi factors rather than to directly regulate heterochromatin assembly through non-coding RNAs. Cwf14 is also required for telomeric heterochromatin assembly as it regulates the proper splicing of telomere shelterin component *tpz1^+^*. As expected, *cwf14Δ* was also identified as a factor required for telomeric heterochromatin assembly in our telomere-silencing screen (Figure 1D).

Another mutant identified in our screen is *sde2Δ*, which was recently characterized as a factor required for telomere silencing (36). Similar to *tls1Δ*, *sde2Δ* has no effect on silencing at pericentric or the mating-type regions (36). Interestingly, Sde2 contains a region that shares sequence homology with splicing factor SF3A60 in humans, and it is consistently identified in purifications of spliceosome components, raising the possibility that it also regulates the proper splicing of certain heterochromatin assembly factors (24,54,57). The molecular function of Sde2 is currently unknown, and it would be interesting to examine whether Sde2 is also a *bona fide* splicing factor and, if so, the mechanism by which it regulates splicing.

The identification of Tls1, which regulates the proper splicing of mRNAs of shelterin components *rap1^+^* and *poz1^+^* to control telomeric heterochromatin assembly, further corroborates the idea that splicing factors affect heterochromatin assembly mainly by regulating the splicing of heterochromatin assembly factors. However, it should be noted that Tls1 is very unique in its function as a splicing regulator. In contrast to Cwf14, which is consistently identified in purification of spliceosome components and is present in comparable amounts to other spliceosome subunits, Tls1 was not identified in purifications of spliceosome components (24,54,57). Purification of overexpressed Tls1 showed high levels of one component of the U5-snRNP, Brr2, whereas other components of the spliceosome were not identified or were found only at extremely low levels (Figure 4C). These results suggest that Tls1 either peripherally or transiently associates with the spliceosome through Brr2.

In accord with the data that Tls1 associates with spliceosome components, RNA sequencing analysis shows that *tls1Δ* cells are defective in splicing of a subset of mRNAs. Unlike *cwf14Δ*, which is a stable component of the core spliceosome required for the splicing of RNAi components, missplicing of which results in pericentric heterochromatin defects, *tls1Δ* cells display no silencing defects at pericentric regions, which is consistent with the fact that RNAi factors are properly spliced in *tls1Δ* cells. Thus, Tls1 seems to be selectively required for the splicing of *rap1^+^* and *poz1^+^* mRNAs. However, comparison of misspliced introns in *tls1Δ* cells did not reveal any common features and the mechanism for this selectivity is unknown.

Rap1 and Poz1 form a molecular link that connects Taz1 and Tpz1-Pot1 complex, which bind to double-stranded and single-stranded portions of telomeric DNA, respectively (6). The proper connection between these proteins is essential for maintaining a state of telomeres refractive to telomerase-mediated telomere lengthening (32). Therefore, Tls1 might provide a novel mechanism for controlling telomere length by disrupting the connection between these two regions, by regulating the proper splicing of the adaptor proteins Rap1 and Poz1.

Tls1 is a conserved protein present in diverse organisms. Its human homologue, C9ORF78, was originally identified as a factor that is overexpressed in hepatocellular carcinoma and a number of other cancer cell lines, but not in their normal tissue counterparts, indicating a possible role of this protein in tumorigenesis (25). However, the molecular mechanism by which this protein functions is completely unknown. Our result showing that Tls1 associates with the spliceosome and that Tls1 regulates the splicing of shelterin in fission yeast suggest that C9ORF78 is likely a splicing factor as well. Indeed, C9ORF78 was present in purifications of mammalian spliceosome components (58–62), suggesting the interaction is evolutionarily conserved. Given the prevalence of splicing misregulation in cancer cells (63), it is interesting to examine whether C9ORF78 also regulates the proper splicing of mRNAs and whether such misregulation underlies tumorigenesis.

## SUPPLEMENTARY DATA

Supplementary Data are available at NAR Online.

SUPPLEMENTARY DATA
