# Supplementary material for: Tls1 regulates splicing of shelterin components to control telomeric heterochromatin assembly and telomere length
Source: Nucleic Acids Res. 2014 Sep 22;42(18):11419–32. doi: 10.1093/nar/gku842 (PMC4191416; doi:10.1093/nar/gku842)
Supplement: SUPPLEMENTARY DATA [file supp_42_18_11419__index.html]

Tls1 regulates splicing of shelterin components to control telomeric heterochromatin assembly and telomere length — Tls1 regulates splicing of shelterin components to control telomeric heterochromatin assembly and telomere length — SUPPLEMENTARY DATA 

# Tls1 regulates splicing of shelterin components to control telomeric heterochromatin assembly and telomere length

## SUPPLEMENTARY DATA

**Files in this Data Supplement:**

- SUPPLEMENTARY DATA
- SUPPLEMENTARY DATA
