# Supplementary material for: Tls1 regulates splicing of shelterin components to control telomeric heterochromatin assembly and telomere length
Source: Nucleic Acids Res. 2014 Sep 22;42(18):11419–32. doi: 10.1093/nar/gku842 (PMC4191416; doi:10.1093/nar/gku842)
Supplement: SUPPLEMENTARY DATA [file supp_gku842_nar-01301-a-2014-File009.pdf]

Figure S1

```
Hs : ---------MPVVRKIFRRR---RGDSESE--EDEQDSEEVRIKLEETREVQNLRKRPNGVSAVALLVGEKVQ-EETTLVDDPFQMKTGGMVDMKKLKERGKDKIS
Mm : ---------MRITGKTFRRR---RADSESE--EDEQESEEVRIKLEETREVQNLRKRPNGVSAAALLVGEKVQ-EETTLVDDPFQMATGGMVDMKKLKERGKDKVS
Xl : ---------MFAG-RNFRRR---KASSSDEEVEDEGVTREVRMKLEEAKEVQSLRKRQNGVSAAALLVGEKLP-EEVNMADDPFKMQNGGMVDMKKLKDRGKDRIG
Dm : MSDAEETKAEATTKVLFKKA---GRKNLRQRKNSDEEEKEEQLTLDEIKERQRLRQRPNGVSLVGIALGKKIAPEEEIAIKDPFNVKTGGLVNMKQLKSGKMKEAD
At : -----MPPKRNFRKRSFEEE---EEDNDVNKAAISEEEEKRRIALEEVKFLQKLRERKLGIPALSSTAQSSIG---------------------KVKPVEKTETEG
Sp : ---MNSIHIKKKSNRSFRRRKVFGNEKEFDLEELDDNDIRLRQALEATK-RRKIRNSIIGINAEKLLNQETKKEKQLNTANEP--------HEANDQTSAQSSKLI

Hs : EEEDLHLGTSFSAETNRRDEDADMMKYIETELKKRKG-----IVEHEEQKVKPKNAED-CLYELPENIRVSSAKKTEEMLSNQMLSGIPEVDLGIDAKIKNIISTE
Mm : EEEDLHLGTSFSAETNRRDEDADMMKYIETELKKRKG-----IVEQEEQKAKPKNAED-CLYELPENIRVSSAKKTEEMLSNQMLSGIPEVDLGIDAKIKNIISTE
Xl : EEEDINLGTSFSAETNRRDEDADMMKYIETELKKRKG-----IVENEEKKVKPKSAED-CLYELPESIKVSSAKKTEEMLSNQMLSGIPEVDLGIDAKIKNIISTE
Dm : DAYDVGIGTQFSAETNKRDEDEEMMKYIEQELQKRKGGGTEDAAEDDGDVNKYLTPEDAALYALPDHLRQSSSHRSEEMLSNQMLNGIPEVDLGIVAKIRNIEATE
At : EKEELVLQDTFAQETAVLIEDPNMVKYIEQEIAKKRG----RNIDDAEEVENELKRVEDELYKIPDHLKVK--KRSSEESSTQWTTGIAEVQLPIEYKLKNIEETE
Sp : EAQLPTVEDRFAKQTNEVDINTHLINFVEKKLKQERIA---QNYSENGETNALNTKNESTVQNIKNSLHPN---EHSFIRDAAAIGAIREVDLGIIS--TDVDNLK
```

HEP59

```
Hs : DAKARLIAEQQNKKKDSETSFVPTNMAVNYVQHNRFYHEELNAPIRRNKEEPKARPLRVGDTEKPEPERSPPNRKRFANEK--ATDDYHYEKFKKMNRRY----
Mm : DAKARLIAEQQNKKKDSETSFVPTNMAVNYVQHNRFYHEELNAPIRRNKEEPKARPLRVGDTEKPEPERSPPNRKRFANEK--ATDDYHYEKFKKMNRRY----
Xl : EAKARLIAEQQNKKKDKHTSFVPTNMAVNYVQHNRFYQEDQNTPMRRHKEEPKPRPLRVGDTEKPEPEKSPPNRKRPSNEK--ATDDYHYEKFKKMNRRY----
Dm : EAKQKLIQDAKNKK-DGPSQFVPTNMAVNFMQHNRFNIEDNSDQRRRKREE------REGN--KSAQHQTNPN----GVKR--ATDDYHYDKFRKQFRRY----
At : AAKK-LIQERRLMGRPKSEFSIPSSYSADYFQRGKDYAEKLRREHPELYKD-RGGPQADGEAAKPSTSSSTNNNADSGKSRQAATDQIMLERFRKRERNRVMRR
Sp : NGRKRQKKRARMKEKLDSKALRTSEDAAR-----DEFIEKMLKPISQDEES------------------------KGIYR-------RFRVYKDGTQD-----
```

Figure S1: Sequence alignment of Tls1 homologues.

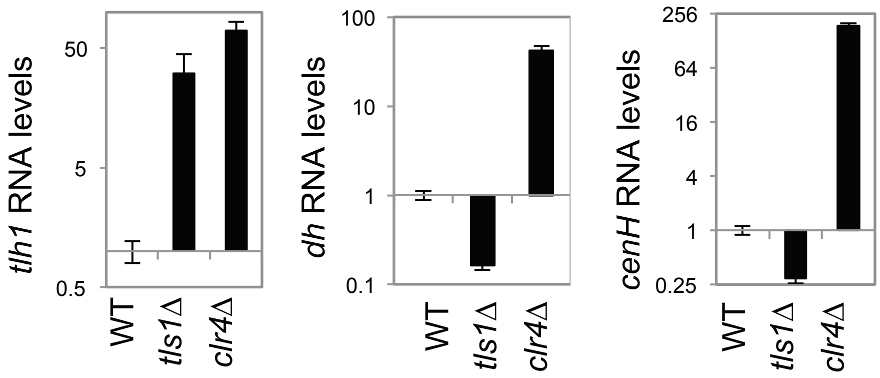

Figure S2: qRT-PCR analyses of transcripts derived from repeat elements, normalized to *act1*. The data is the same as Fig. 2C, but plotted in log scale to show the down regulation of *dh* and *cenH* RNAs.

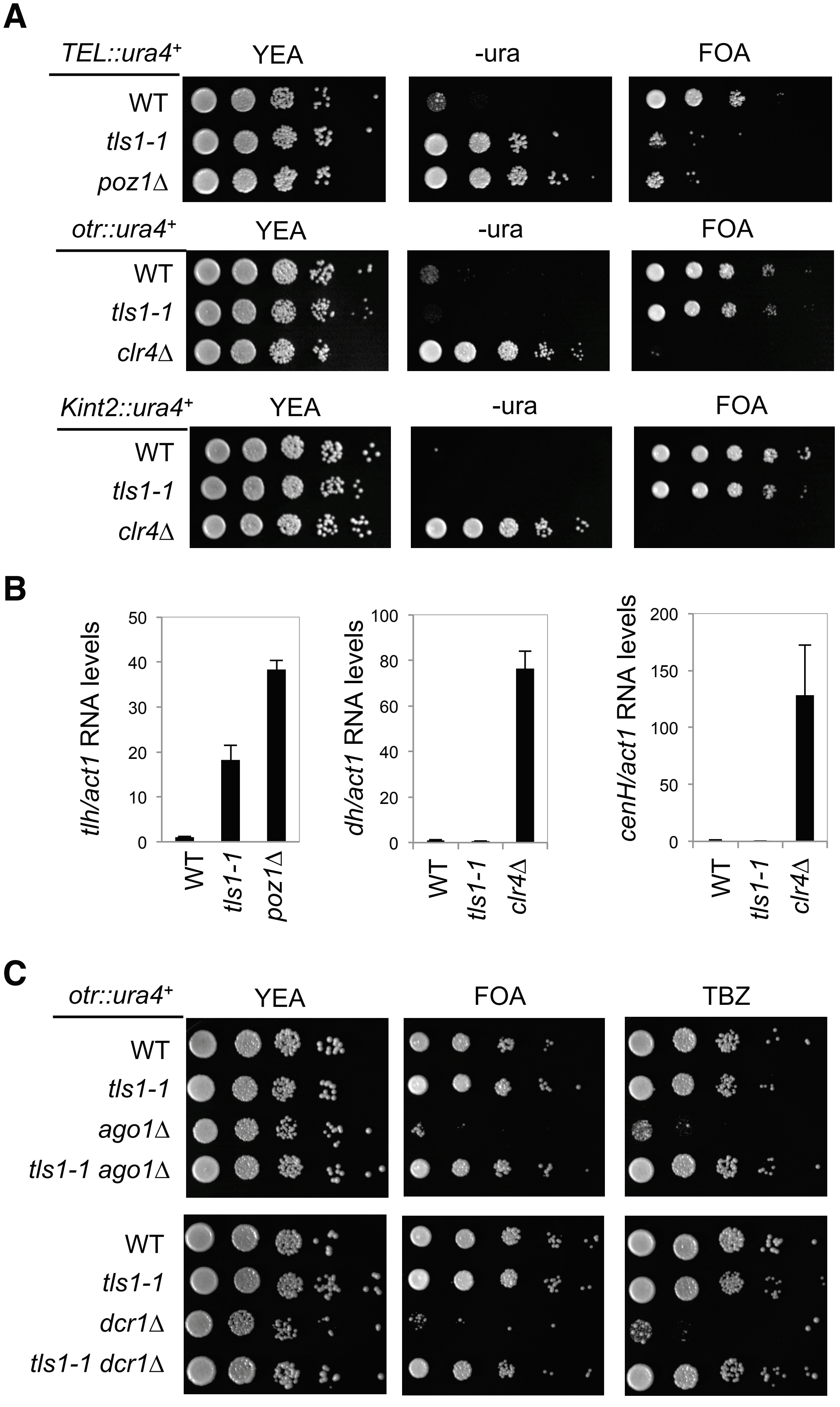

Figure S3: Characterization of *tls1-1*. (A and C) Serial dilution analyses to measure the expression of *ura4*+ reporter genes and sensitivity to TBZ. (B) qRT-PCR analyses of transcripts derived from repeat elements, normalized to *act1*.
